# Mucosal gene therapy using a pseudotyped lentivirus vector encoding murine interleukin-10 (mIL-10) suppresses the development and relapse of experimental murine colitis

**DOI:** 10.1186/1471-230X-14-68

**Published:** 2014-04-08

**Authors:** Hiroshi Matsumoto, Kazunori Haga, Izumi Ohno, Kei Hiraoka, Takahiro Kimura, Kip Hermann, Noriyuki Kasahara, Peter Anton, Ian McGowan

**Affiliations:** 1 Department of Medicine, Division of Digestive Diseases, David Geffen School of Medicine at the University of California (UCLA), Los Angeles, CA, USA; 2 Department of Molecular & Medical Pharmacology, David Geffen School of Medicine at the University of California (UCLA), Los Angeles, CA, USA; 3 Magee-Womens Research Institute, Division of Gastroenterology, Hepatology and Nutrition, University of Pittsburgh School of Medicine, Pittsburgh, PA, USA; 4 Division of Gastroenterology and Ultrasonography and Translational Medical Science and Nutrition (GUT), Kawasaki Medical School, Okayama, Japan; 5 Division of Gastroenterology, East Hospital of National Cancer Institute, Chiba, Japan; 6 Division of Thoracic Surgery, Hokkaido University, Hokkaido, Japan; 7 Division of Urology, Jikei University School of Medicine, Tokyo, Japan

**Keywords:** Gene therapy, Colitis, Lentivirus vector, Interleukin-10

## Abstract

**Background:**

Therapeutic gene transfer is currently being evaluated as a potential therapy for inflammatory bowel disease. This study investigates the safety and therapeutic benefit of a locally administered lentiviral vector encoding murine interleukin-10 in altering the onset and relapse of dextran sodium sulfate induced murine colitis*.*

**Methods:**

Lentiviral vectors encoding the reporter genes firefly-luciferase and murine interleukin-10 were administered by intrarectal instillation, either once or twice following an ethanol enema to facilitate mucosal uptake, on Days 3 and 20 in Balb/c mice with acute and relapsing colitis induced with dextran sulfate sodium (DSS). DSS colitis was characterized using clinical disease activity, macroscopic, and microscopic scores. Bioluminescence optical imaging analysis was employed to examine mucosal lentiviral vector uptake and transgene expression. Levels of tumor necrosis factor-α and interleukin-6 in homogenates of rectal tissue were measured by ELISA. Biodistribution of the lentiviral vector to other organs was evaluated by real time quantitative PCR.

**Results:**

Mucosal delivery of lentiviral vector resulted in significant transduction of colorectal mucosa, as shown by bioluminescence imaging analysis. Lentiviral vector-mediated local expression of interleukin-10 resulted in significantly increased levels of this cytokine, as well as reduced levels of tumor necrosis factor-α and interleukin-6, and significantly reduced the clinical disease activity, macroscopic, and microscopic scores of DSS colitis. Systemic biodistribution of locally instilled lentiviral vector to other organs was not detected.

**Conclusions:**

Topically-delivered lentiviral vectors encoding interleukin-10 safely penetrated local mucosal tissue and had therapeutic benefit in this DSS model of murine colitis.

## Background

Inflammatory bowel disease (IBD), comprising Crohn’s disease (CD) and ulcerative disease (UC), is thought to result from abnormal interactions between gut associated lymphoid tissue and enteric microflora. This abnormal mucosal immune response is probably facilitated by defects in epithelial barrier function [1]. Interleukin (IL)-10 plays a crucial role in mucosal immunoregulation, inhibiting aspects of both the innate and cell mediated inflammatory response [2]. IL-10 has broad immunoregulatory activity and acts to suppress intestinal inflammation on several levels. Gene-targeted IL-10 knockout mice (IL-10−/−) and IL-10 receptor 2 deficient mice spontaneously develop an enterocolitis by 2–3 months of age with multifocal inflammatory lesions throughout the gastrointestinal tract [3]. In addition, mutations in the gene encoding the IL-10 receptor subunit proteins have been found in IBD patients [4]. IL-10 suppresses the release of many other proinflammatory cytokines and chemokines, including tumor necrosis factor alpha (TNF-α), IL-1, IL-6, and IL-8. Finally there is strong evidence that IL-10 acts to promote the differentiation and augment the activity of regulatory T cells [5].

Administration of systemic IL-10 is sufficient to inhibit inflammation and abrogate experimental colitis models [6,7]. However, clinical trials in Crohn’s disease have shown that, although daily systemic IL-10 injections are safe and well tolerated, they have minimal therapeutic efficacy [8,9]. One explanation for these disappointing results is that daily systemic therapy does not deliver adequate mucosal levels of IL-10 that would be needed to control proinflammatory responses associated with active CD. Thus, strategies that result in greater mucosal exposure to IL-10 in the gastrointestinal tract may prove effective in treating IBD.

Several IL-10 delivery systems targeting the gastrointestinal mucosa have been reported; rectal administration using an adenovirus vector [10], oral delivery using non-pathogenic bacteria (*Lactococcus lactis*) [11], and oral nanoparticles [12]. Recently, we demonstrated the ability of a vesicular stomatitis virus envelope glycoprotein (VSV-G) pseudotyped lentiviral vector (LV) to infect colonic mucosal tissue via the apical surface *in vivo,* after intraluminal instillation per rectum in a healthy murine model and *ex vivo* in a human intestinal explant system [13]. In this study, we tested whether efficient local gene transfer can be accomplished using LV expressed murine IL-10 (mIL-10) in a murine colitis model.

## Methods

### Vector construction and preparation

The pRRLsin-hCMV-fLuc vector was constructed by insertion of the firefly Luciferase (fLuc) gene [14], and the pRRLsin-hCMV-mIL10 vector by insertion of the mIL-10 coding sequence from plasmid pORF5-mIL-10 (InvivoGen, San Diego, CA, USA), respectively, into the multiple cloning site (MCS) of pRRLsin-hCMV-MCS-pre, a third-generation, self-inactivating LV construct provided by Dr. Luigi Naldini (San Rafaelle Telethon Institute, Milan, Italy) [15]. All constructs were reconfirmed by restriction digestion and DNA sequencing analysis. Vesicular stomatitis virus glycoprotein envelope (VSV-G)-pseudotyped LV virus was produced in 293 T cells using a third-generation packaging system as previously described [16]. LV titers were determined by HIV-1 p24 ELISA (Coulter Immunotech, Miami, FL, USA) and expressed as p24 equivalent units (ng/ml).

### Cell culture *in vitro* gene transfer study

Three human colonic cancer cell lines of epithelial origin (CaCo-2, LoVo, and WiDr) were obtained from the American Type Tissue Collection (ATCC, Manassas, VA, USA) and grown at 37°C in 5% CO_2_ in Dulbecco’s modified Eagle’s medium, Ham’s F12K medium, or RPMI 1640, respectively. All media were supplemented with 10% fetal bovine serum and 1% penicillin-streptomycin. Transductions were done using media containing polybrene (8 μg/ml) (Sigma, St. Louis, MO, USA) to enhance gene transduction [17]. Each transduction was carried out with 1 × 10^5^ cells. To assess the mIL-10 productive capacity of the LV constructs, 100 ng p24 of the LV-mIL-10 (equivalent to a biological infectious titer of 5 × 10^6^ transducing units on the standardized cell line HEK-293 T [18]) was added to monolayer cultures of the three specified cell lines at a multiplicity of infection (MOI) of 0.1, 1.0 or 10 (ratio of virus to cell number). Transduction was performed with the viral solution at 37°C for 12 hours followed by a change of medium and a second 24 hour incubation. After the 36 hour transduction period, supernatants were collected, filtered, and stored for batch quantification of mIL-10 level by ELISA.

### Preliminary treatment to enhance mucosal delivery of the LV

Mucosal pretreatment with a 20% ethanol (EtOH) enema was used to enhance gene delivery into the colonic mucosa. Female Balb/c mice (weight: 19–23 grams, age: 6–8 weeks) (Charles River Laboratories, Inc., Wilmington, MA, USA) were housed in a specific pathogen-free environment prior to LV exposure. All *in vivo* studies were performed under the appropriate guidelines and with the approval of the UCLA Animal Research Committee.

### *Ex vivo* bioluminescence imaging

*Ex vivo* bioluminescence imaging (BLI) of transduced tissue from gastrointestinal (GI) organs was used to assess LV-guided gene transfer into the colonic mucosa. Two days following topical exposure to a LV expressing fLuc, GI tract organs from each group were harvested *en bloc*, bathed and imaged using a cooled charge-coupled device (CCD) system (Xenogen IVIS, Caliper Life Sciences, Alameda, CA, USA) where gray-scale background photographic images of the tissues were overlaid with color images of bioluminescent signals (Living Image and IGOR-PRO image analysis software, Wave Metrics, Portland, OR, USA).

### LV-mIL-10 treatment effect on dextran sulfate sodium colitis

Colitis was induced by exposure to 3% (w/v) dextran sulfate sodium (DSS; molecular weight 36-50 kDa; ICN Pharmaceutical, Costa Mesa, CA) administered orally which reproducibly produces histologic inflammation mainly in the left colon [19,20]. Each mouse received 2 cycles of DSS oral treatment with each cycle consisting of 7 continuous days with DSS added to the drinking water followed by a 10 day period without DSS.

For this experimental design, mice were divided four groups (n = 8 mice/group): (i) non-exposed, healthy controls (NC), (ii) mock-exposed (plasmid complex solution without the mIL-10 vector), (iii) once-exposed treatment group (LV(1)) and (iv) twice-exposed treatment group (LV(2)). LV-mIL-10 was given on day 3 of each of the two DSS cycles. Mice were anesthetized with an intraperitoneal injection of 10 mg/kg xylazime and 100 mg/kg ketamine. Two hours following the preliminary EtOH enema treatment, 100 μl of 1000 ng/p24 viral solution was atraumatically injected intrarectally via a 1.2 mm diameter catheter. Mice were sacrificed on Day 10 (n = 4) or 32 (n = 4), following each DSS exposure.

### Clinical, macroscopic and histologic assessments

The health status of all treated mice was followed closely and all mice were weighed 3 times/week. The clinical disease activity score (CDA; scores range from 0–4) was assessed using a previously published index incorporating body weight, stool consistency and occult blood [19].

Postmortem, the entire colon was removed from the cecum to the anus, flushed with saline, and placed without tension on cellulose [19,20] where it was fixed in 4% paraformaldehyde overnight. Subsequently, the tissue was placed in 30% sucrose/PBS for 2 hrs, embedded in OCT compound with serial 5.0-μm-thick frozen sections sliced from the block and stained with hematoxylin and eosin (H&E) for histologic scoring (range: 0–6) by a pathologist, blinded to sample groupings. Macroscopic colonic damage score was assessed during colon removal as previously reported [21] with minor modifications. The scoring, with ranges from 0–9, included scales for degrees of tissue adhesion, presence of ulceration, and wall thickness. A combined score was used next to assess injury from the macroscopic and the histologic grading [22]. The scores for cell infiltration and tissue damage were added, resulting in a combined histologic score ranging from 0 to 6.

### Cytokine production

As described previously, tissue was homogenized in nine volumes of Greenberger lysis buffer (300 mM NaCl, 15 mM Tris HCl, 2 mM MgCl2, 2 mM, Triton (X-100), pepstatin A, leupeptin, aprotinin (all 20 ng/ml), pH 7.4) [23,24]. Tissue was lysed for 30 min on ice followed by two centrifugations at 14,000 g for 10 min each. Homogenates were stored at −20°C until further use. TNF-α, IL-6 and IL-10 protein concentrations (pg/mL) were measured by ELISA (R&D Systems, Abingdon, England).

### Biodistribution analysis by real-time quantitative PCR (RT-qPCR)

To determine the lentiviral DNA copy number within the colorectal tissues following topical exposure, we performed qPCR. Genomic DNA was isolated from mouse colonic tissue using DNeasy Tissue Kit (Qiagen, Inc., Valencia, CA, USA). Quantification of vector copy numbers was performed in 25 μL reactions containing 300 ng genomic DNA (equivalent to 5 × 10^4^ genomes) using TaqMan qPCR assay to detect the HIV-1 packaging sequences as a universal primer [25]. Amplifications were carried out in an ABI PRISM 7700 sequence detector (Perkin Elmer, Wellesley, MA, USA). After the initial denaturation step (10 min at 95°C), amplification was performed with 40 cycles of 15 s at 95°C, and 60 s at 60°C. To calculate the copy number in the samples, a reference curve was prepared by amplifying serial dilutions of a LV-CMV-Luc plasmid in a background of genomic DNA obtained from untransduced murine colon. Genomic DNA from the PC3 cell line, a human prostate cancer cell line, transduced with LV-GFP was used as a positive control. PC3 cells were shown to have 100% transduction by flow cytometry and as these cells are hyperploid, the vector copy number in 300 ng of PC3 genomic DNA was quantitated as approximately 16,000 (data not shown).

### Statistical analysis

All values were expressed as means ± SD. Comparisons between groups were made using the student *t*-test and the Mann–Whitney *U* test. A *p* value of less than 0.05 was considered statistically significant. Statistical analysis was performed using Graph Pad Prism (Version 4.00 for Macintosh; GraphPad Software, San Diego, CA, USA).

## Results

### IL-10 production *in vitro*

**Figure 1:**
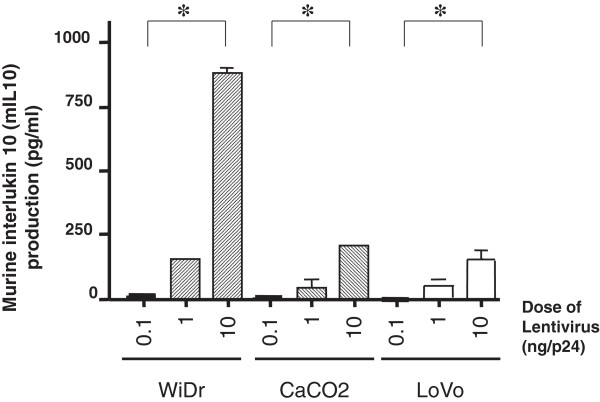
**Quantification of *in vitro *transduction of vesicular stomatitis virus G protein (VSV-G)-pseudotyped lentivirus (LV) encoding mouse interleukin 10 (mIL10) in colonic cell lines.** Cell line production of mIL-10 in pg/ml following three titered infectious doses of LV (0.1 ng/p24, 1.0 ng/p24, 10 ng/p24) for transfection are shown. Each experiment collected took triplicate supernatant, and measured IL-10 by ELISA. All values were expressed as mean ± SD. *p < 0.05 compared with the results of 0.1 ng p24 LV transduction.

The level of IL-10 production following transduction with the mIL-10 LV was 881 pg/ml in WiDr cells, 205 pg/ml in CaCo-2 cells, and 153 pg/ml in LoVo cells. All cell lines produced measurable mIL-10, with expression levels increasing in a vector dose-dependent manner (Figure 1).

### Bioluminescent imaging of LV transfer

**Figure 2:**
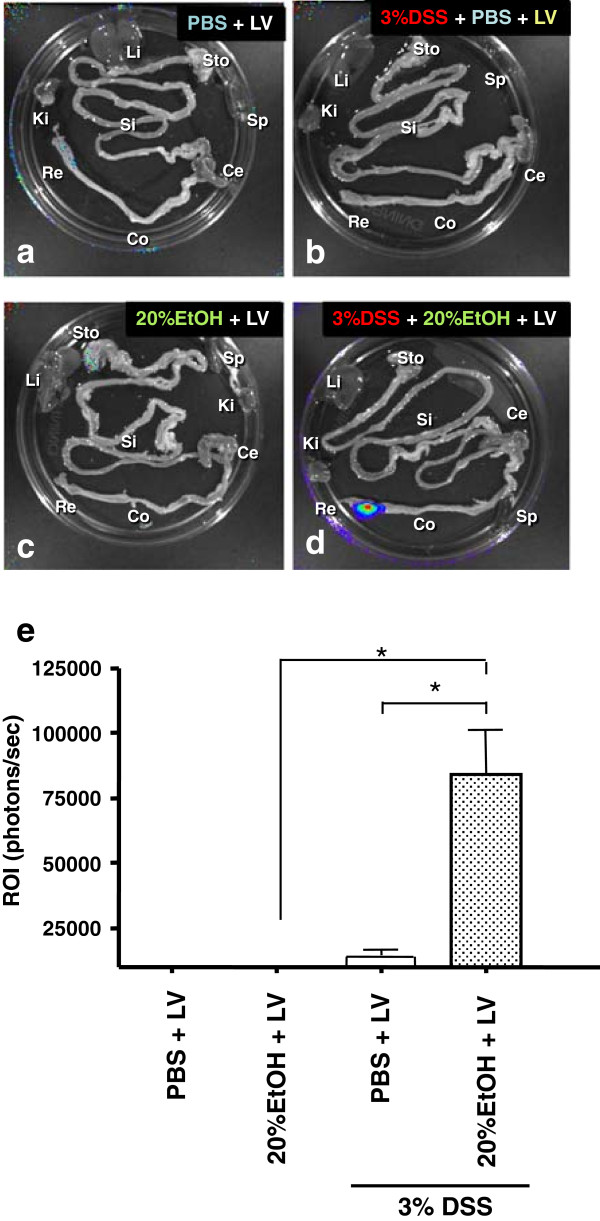
**Optimization of mucosal LV transfection. ***Ex vivo* Bioluminescence imaging (BLI) analyses. *Ex vivo* BLI quantification and analyses of GI tract following intrarectal administration of LV expressing firefly luciferase (fLuc) on either colon with **(a)** PBS only, **(b)** with PBS followed by 3% DSS, **(c)** with 20% EtOH only, or **(d)** with 20% EtOH followed by 3% DSS. This pseudocolor image, superimposed on a gray scale reference image, uses color (blue: least intense; red: most intense) to illustrate signal strength. *Abbreviations: Sto: stomach; Si: small intestine; Co: colon; Ce: Cecum; Re: rectum; Li: liver; Ki: kidney; Sp: spleen*. **(e)** quantifies photon emission (p/s/cm2/sr) in the distal colon. *p < 0.05.

BLI analysis of the entire removed colon was used to quantify the regional distribution and levels of gene transduction by LV encoding fLuc following various delivery protocols. A strong positive signal was seen only in the distal colon, adjacent to the rectum, in 8/8 (100%) of the mice exposed to 20% EtOH plus LV in the 3% DSS colitis model (Figure 2a-d). Significantly higher bioluminescent light signals were observed after LV delivery with 20% EtOH than with saline (PBS) control pre-treatment in the 3% DSS colitis groups, indicating a significantly higher level of transduction with ethanol pre-treatment (91,260 ± 23,060 versus 8881 ± 1655 photons/sec) (Figure 2e).

### Impact of LV mIL-10 gene therapy on DSS colitis severity

**Figure 3:**
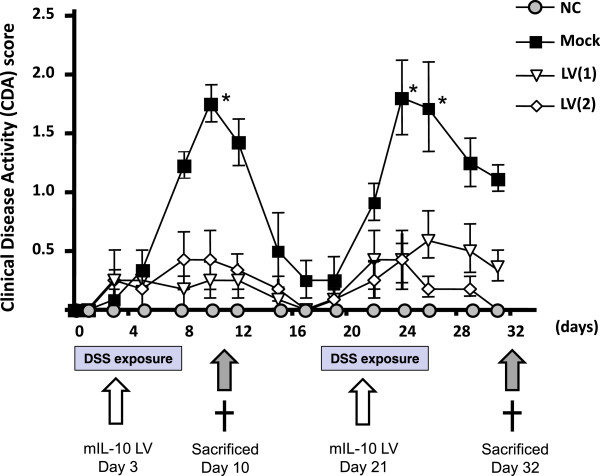
**The time course of clinical changes of 3% dextran sulfate sodium (DSS) colitis with/without lentivirus vector (LV) encoding mouse interleukin 10 (mIL-10).** Mice were treated with 3% DSS from Days 1–7 and 18–24. Four treatment groups (n = 8 mice per group) were (i) a control group who did not receive DSS or an LV (NC), (ii) a group who received DSS + a mock plasmid control (mock), (iii) a group who received DSS + a single dose of the mIL-10 LV on Day 3 (LV(1)), and (iv) a group who received DSS + doses of the mIL-10 LV on Days 3 and 20 (LV(2)).

**Figure 4:**
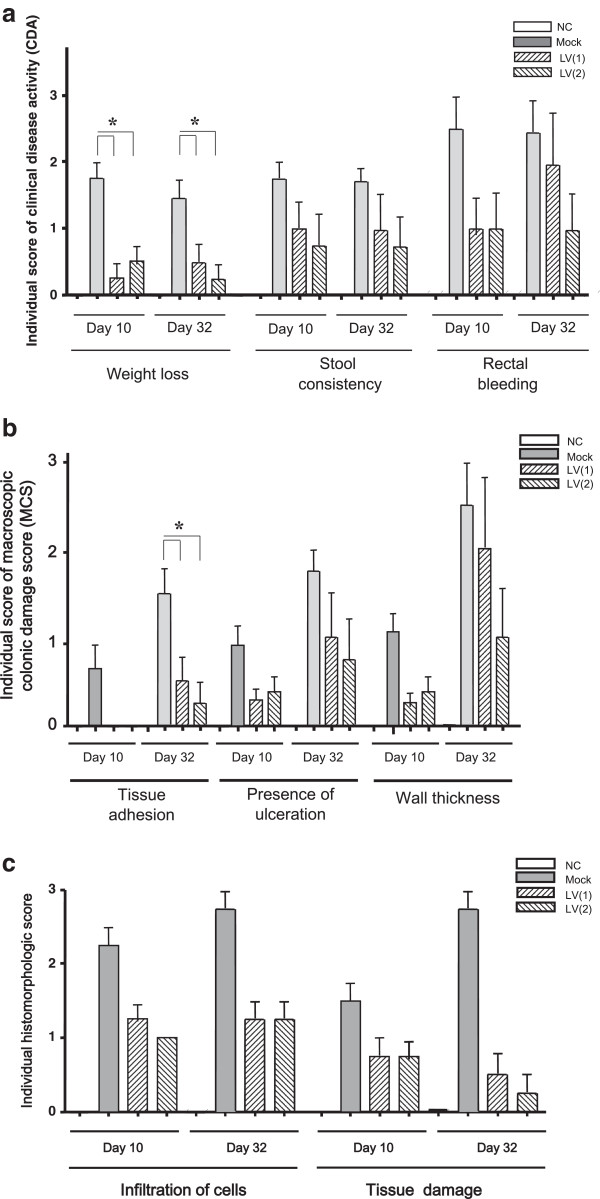
**Clinical, macroscopic, and histopathological changes following treatment with lentivirus vector encoding mouse interleukin 10 (mIL-10). (a)** CDA score was monitored 3 day per a week. Each point represents the mean ± SD. Balb/c mice received Mock or LV encoding mIL-10 (LV mIL-10) once or twice by rectal administration. There is a significant difference between Mock and both LV mIL-10 (1) and (2), however no difference between LVmIL-10(1) and (2) on Days 10 and 24. All values were expressed as mean s ± SD. Statistically significant differences are shown. *P < 0.05. **(b)** Changes in macroscopic colonic damage scores of 3% DSS colitis treated with LV encoding mIL-10. Macroscopic colonic damage score of mock-treated, LV once-treatment/DSS exposure group (LV(1)) or LV twice-treatment/DSS exposure group (LV(2)) compared to no treatment groups (n = 8/group) are shown for each of the two DSS exposures on Day 10 or Day 32. All values were expressed as mean ± SD. Statically significant differences are shown. *p < 0.05. **(c)** Changes in histologic scoring of 3% DSS colitis treated by LV encoding mIL-10. The degree of histological injury following DSS exposure on Day 10 and Day 32 are shown for mock-treated, LV once-treatment/DSS exposure group (LV(1)) or LV twice-treatment/DSS exposure group (LV(2)) compared to no treatment groups (n = 8/group). All values were expressed as mean ± SD. Statically significant differences are shown. *p < 0.05.

**Figure 5:**
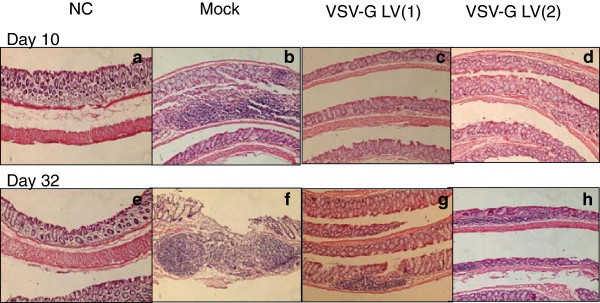
**Histopathology sections of DSS colitis treated with LV encoding mIL-10.** Representative sections of each treatment groups are shown following DSS exposure at Day 10 **(a, b, c, d)** or Day 32 **(e, f, g, h)**. Normal controls for each are shown in **(a)** and **(e)**. Mock-treatment group showed the expected severe colonic inflammation on both Days 10 and 32. Treatment with LV encoding mIL-10, regardless of whether given once per DSS cycle (LV(1)) or twice/cycle (LF(2)), showed reduction in inflammation on both Days 10 and 32. (H & E stain; magnification: 200×).

DSS-induced mucosal injury was quantified by clinical, macroscopic and histologic scores. All three indices were significantly improved following exposure to LV producing mIL-10. Figure 3 shows the time course of clinical disease activity (CDA) by study arm. The mock exposed group showed two peaks of severity using the CDA score, on Days 9 and 24. LV gene therapy significantly suppressed both of these peaks overall as well as in each of the sub-categories (weight loss, stool consistency, rectal bleeding) (Figure 4a). Macroscopic (tissue adhesion, ulceration, wall thickness) (Figure 4b) and histological (infiltration of cells, tissue damage) (Figure 4c, Figure 5a-h) scores were also each significantly suppressed by LV gene therapy compared to the mock treated mice.

### Changes in mucosal cytokine proteins in DSS colitis tissue treated by LV mIL-10

Neither IL-10 nor IL-6 were detectable in the colonic homogenates from the NC mice. TNF-α was present at low levels (0.7 ± 0.3 pg/ml at Day 10 and 0.6 ± 0.4 pg/ml at Day 32). In contrast, all three cytokines were present in the mock-treated group of mice, which were exposed to DSS but did not receive LV-mediated gene therapy (Table 1). Mice receiving one or two exposures to the IL-10 LV had significantly higher mucosal tissue levels of IL-10 than either the control or mock-treated groups. In mice receiving one exposure to the IL-10 LV, levels of IL-10 were 18.1 ± 3.9 pg/ml at Day 10 and 12.9 ± 12.1 pg/ml at Day 32. In the mice receiving two exposures to the IL-10 LV, levels of IL-10 were 15.9 ± 3.7 pg/ml at Day 10 and 14.3 ± 0.7 pg/ml at Day 32. Interestingly, TNF-α was reduced in all the mice receiving the IL-10 LV. In mice receiving one exposure to the IL-10 LV, levels of TNF-α were 11.5 ± 5.4 pg/ml at Day 10 and 3.5 ± 1.4 pg/ml at Day 32. In the mice receiving two exposures to the IL-10 LV, levels of TNF-α were 3.9 ± 1.8 pg/ml at Day 10 and 3.9 ± 2.9 pg/ml at Day 32. IL-6 was undetectable in all of the mice receiving the IL-10 LV.

**Table 1:** Cytokine levels in mucosal tissue

**Cytokine**	**DSS day**	**NC**	**Mock**	**LV(1)**	**LV(2)**
**IL-10**	10	ND	3.9 ± 1.6	18.1 ± 3.9*	15.9 ± 3.7*
	32	ND	ND	12.9 ± 12.1	14.3 ± 0.7
**TNF-α**	10	0.7 ± 0.3	25.4 ± 9.4	11.5 ± 5.4	3.9 ± 1.8
	32	0.6 ± 0.4	15.2 ± 6.5	3.5 ± 1.4*	3.9 ± 2.9
**IL-6**	10	ND	ND	ND	ND
	32	ND	14.5 ± 1.3	ND	ND

*p <0.05 compared to mock infection.

ND; Not detected.

### Biodistribution study by qPCR analysis

**Figure 6:**
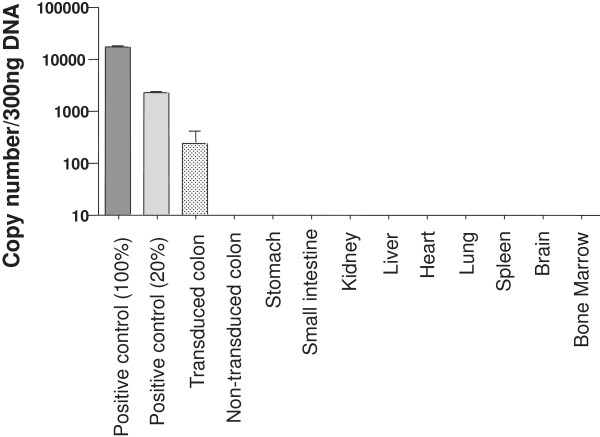
**Analysis of LV biodistribution by real-time quantitative PCR (RT-qPCR).** Genomic DNA was isolated from mouse colonic tissue and vector copy number was quantified using TaqMan qPCR assay to detect the HIV-1 packaging sequences. Genomic DNA from the PC3 cell line, a human prostate cancer cell line, transfected with LV-GFP was used as a positive control.

Biodistribution analysis by qPCR showed evidence of transduction in the colorectum in all mice treated with 3% DSS upon local LV administration following 20% EtOH (Figure 6) but in no other areas of the gastrointestinal tract (rest of colon, small intestine, stomach or liver). Transduction levels in the colorectum were on the order of 2 to 3%. No gene expression was identified in any extra-gastrointestinal tissue examined (kidney, spleen, lung, heart, brain, bone marrow). These data confirm that topical administration of LV enabled local tissue transduction but minimal spread to other regions within the gastrointestinal tract, and no detectable extra-intestinal spread.

## Discussion

In this study, we have demonstrated for the first time that an LV could be used as a novel gene delivery system for the treatment of DSS colitis. Topical gene therapy using a LV encoding mIL-10 was found to suppress not only the development but also the relapse of the colitis. The LV platform is efficient and provides a unique gene transfer system with a number of features that differentiate it from other topical gene therapy approaches that have been evaluated for the treatment and prevention of experimental models of colitis.

Adenovirus vectors [10,24,26-29] and cationic lipids complexed to immunoregulatory genes [30], have reported efficacy in the treatment of murine colitis. However, a major disadvantage of these systems is that the transgene expression is transient since genes are transduced into mucosal epithelial cells that only live for about 2–3 days. Alternatively, an approach using *Lactococcus lactis* only transiently increased mucosal IL-10 concentration because the bacteria did not colonize the intestine [11,31]. *Ex vivo* retroviral gene transfer of CD4+ T cells with IL-10 did not have any therapeutic benefit in acute colitis of immunocompetent mice, though it did prevent induction of colitis in a transfer colitis model using immunodeficient mice [32].

Here we chose to evaluate whether a LV encoding IL-10 was effective in treating or preventing DSS induced murine colitis for three reasons. Firstly, this colitis model induces profound epithelial barrier disruption that might facilitate LV transduction and access to the lamina propria [19]. Secondly, this DSS colitis model shows severe inflammation in the distal colon [19,20,22], the area known to have maximal LV transduction. Finally, mucosal CD4+ cells, an important target of LV transduction, also play a critical role in the pathogenesis of IBD colitis [33]. Within the DSS model, increases in mucosal CD4+ T cells occur in a dose dependent fashion such that with 5% DSS induced colitis is associated with significantly more CD4+ T cells compared to 3% DSS colitis [34]. Supporting our hypothesis, LV expressed mIL-10 showed efficacy against acute and relapsing murine DSS colitis.

Notably, we found that topical delivery of a LV encoding IL-10 could suppress not only the development but also prevent relapse of DSS colitis. This is a unique feature of the LV approach and has not been seen in other traditional vector systems. We suggest that the reason for this property might relate to the ability of the LV to transduce both colonic mucosal epithelial and mucosal CD4+ T cells following rectal administration [13]. Mucosal CD4+ T cells play a dominant role in controlling gastrointestinal mucosal inflammation, especially regulatory CD4+ T cells through secretion of anti-inflammatory cytokines such as IL-10 [5,7]. Interestingly, although not statistically significant, administration of two vector doses showed a trend toward achieving more therapeutic efficacy than a single dose. In this context, it is notable that IL-10 levels were comparable between the single dose and double dose groups, perhaps due to feedback down regulation of endogenous IL-10 in response to vector-derived IL-10. Alternative explanations might include the induction of antibodies or other immunological processes that reduce the efficiency of lentiviral infection and or biological availability of IL-10 in the mucosal tissue. Additional studies are warranted to examine whether higher vector concentrations or frequency of administration might achieve further benefit.

In addition to the barrier disruption caused by DSS colitis, 20% ethanol pre-treatment was also required for optimal LV transduction, perhaps by helping to dissolve or wash away physical barriers obstructing vector access to the cellular surfaces of the mucosal epithelium. While 20% ethanol pre-treatment in itself induces no mucosal inflammation, in the presence of 3% DSS-induced colitis we obtained transduction levels comparable to those observed in our previous studies using 50% ethanol pre-treatment to enhance vector transduction efficiency in the absence of pre-existing mucosal inflammation [13]. Furthermore, similar approaches have been tested both pre-clinically and in clinical trials, e.g., using ethanol or surfactants to improve transduction efficiency of vectors instilled into the bladder, and so this approach has potential for clinical translation in IBD.

There are a number of limitations to our study. Use of additional control groups such as an IL-10 plasmid or lentiviral vector without the IL-10 insert could have provided insight as to the independent contribution of the lentiviral vector and the IL-10 on the subsequent modulation of mucosal infection. In addition we did not measure plasma IL-10 or other immunological parameters that could have provided explanations as to why a second dose of the LV did not appear to provide benefits above or beyond those seen with a single dose of LV.

## Conclusion

In conclusion, we have demonstrated that topical gene therapy using a VSV-G LV vector to deliver therapeutic levels of IL-10 could have promise as a novel therapy for IBD colitis. Follow-up studies are needed to determine whether this vector system would be effective in treating other models of murine colitis such as the IL-10 knock out model of murine colitis [3].

## Competing interests

The authors declare that they have no competing interest.

## Authors’ contributions

HM conducted the majority of the experiments described in this paper. TK carried out the molecular genetic studies, made the lentiviral vector, and performed the statistical analysis of the experimental data. KH, IO, KH helped to analyze the *in vivo* animal data. NK provided technical assistance for the lentiviral vector experiments, PA collected the endoscopic biopsies, and IM supervised the research, and edited the final manuscript. All authors read and approved the final manuscript.

## Pre-publication history

The pre-publication history for this paper can be accessed here:

http://www.biomedcentral.com/1471-230X/14/68/prepub
